# A validated CT-based scoring system for lateral compression type one pelvic ring injuries provides insight into the spectrum of injury severity and guides treatment decisions; a prospective study

**DOI:** 10.1007/s00590-025-04619-4

**Published:** 2026-01-22

**Authors:** Camryn C. Therrien, Kaj ten Duis, Hester Banierink, Cyril Mauffrey, Jean-Paul P. M. de Vries, Inge H. F. Reininga, Frank F. A. IJpma

**Affiliations:** 1 Department of Trauma Surgery, University of Groningen, University Medical Center Groningen, Groningen, Netherlands; 2 Department of Orthopedic Surgery, Denver Health Medical Center, Denver, USA; 3 Department of Surgery, University of Groningen, University Medical Center Groningen, Groningen, Netherlands

**Keywords:** Pelvic ring injury, Lateral compression type 1, Radiographic scoring system, Patient-reported outcome

## Abstract

**Purpose:**

To gain insight into the spectrum of injury severity in lateral compression type 1 (LC1) pelvic ring injuries using a validated CT-based scoring system and to determine how injury severity relates to treatment and clinical outcomes.

**Methods:**

A prospective study was performed in 203 patients presenting with LC1 injuries at a level one trauma center. CT-scans were assessed using a CT-based scoring system that quantifies injury severity on a scale of 5–14. Patients were categorized into three injury severity groups: low (scores 5–6), intermediate (scores 7–9), and high (scores 10–14) subgroups based on level of sacral displacement, Denis classification, sacral column involvement, inferior ramus displacement, and superior ramus fracture location. Clinical outcomes included delayed intervention due to mal- of non-union, Dutch Short Musculoskeletal Function Assessment and EuroQol-5D 5L at one-year follow-up. Normative data was used to determine recovery.

**Results:**

All patients with low scores (n = 36), 94% of intermediate scores (n = 99), and 71% of high scores (n = 44) were treated conservatively. No conservatively treated patients required delayed intervention. In all subgroups, most recovered to the level of the normative data, with no significant differences in outcomes between operatively and conservatively treated patients.

**Conclusions:**

The LC1 CT-based scoring system provides insight into the spectrum of injury severity and helps guide treatment decisions for LC1 injuries. Patients with low and intermediate injury severity, determined by degree of sacral and pubic rami involvement, can be treated nonoperatively. Those with high injury severity can be treated either conservatively or operatively.

**Supplementary Information:**

The online version contains supplementary material available at 10.1007/s00590-025-04619-4.

## Introduction

Lateral compression type 1 (LC1) injuries, accounting for 57% of pelvic ring injuries [1], are caused by lateral force, resulting in disruptions to both the posterior and anterior pelvic ring [1–6]. LC1 injuries are characterized by a sacral fracture without vertical instability, along with an ipsilateral, contralateral or bilateral fracture of the pubic rami [4, 7, 8]. Within this classification, a substantial range of severity of displacement and instability can occur [6, 7, 9]. Due to the heterogeneity of these injuries, consensus on optimal treatment is lacking [10–14]. Some studies suggest that patients with LC1 injuries can recover well with conservative treatment [15–20], while others argue that these injuries should be operated on to enhance recovery and improve patient outcomes [21–24].

Recently, a validated radiographic scoring system, based on radiographic morphology, was developed by Beckmann et al. to assess the severity of the fracture pattern and guide treatment decisions for LC1 injuries [25, 26]. The scoring system ranges from 5 to 14 and serves as an indicator of the magnitude of pelvic instability. The radiographic parameters assessed in the scoring system can be found in Table 1. A survey of Orthopedic Trauma Association members [25] indicated good predictability of the scoring system on the treatment of patients with low (scores 5–6) and high scores (scores 10–14); however, it showed a lack of consensus in the intermediate scores (scores 7–9). Injuries with intermediate scores require further examination to determine an appropriate treatment. Additionally, the scoring system has not been validated in a clinical setting with patient outcomes [2, 5]. Consequently, the application of the scoring system to a patient population is necessary before implementation in clinical practice. Furthermore, it has been indicated that radiographic elements may predict the likelihood of displacement; however, the relation to patient-reported outcomes remains unknown [27–29]. Therefore, determining the relationship between the radiologic aspects included in the scoring system and patient-reported functional status and health-related quality of life of patients with LC1 injuries is an important next step, providing valuable insight into injury severity and helping guide treatment decisions.

The primary research questions of this study were: 1) How were the treatment decisions made related to the scoring system?; and 2) How were patients with intermediate scores (scores 7–9) treated?. The secondary research questions were: 1) What were the patient outcomes in terms of delayed intervention due to mal- or nonunion, patient-reported functional status and health-related quality of life in patients with low, intermediate or high scores, who were treated operatively or conservatively?; and 2) was there a relation between components of the LC1 scoring system and patient outcome?

## Methods

### Participants

Between 2017 and 2024, a prospective study was performed in a level one trauma center, including all consecutive adult patients presenting with a pelvic injury [30]. Patients without known cognitive disorders and those able to communicate in Dutch were invited to complete questionnaires at several follow-up moments [30]. The local Medical Ethical Review Board assessed the study methods and waived the need for further approval (METc 79 2017/543).

Upon clinical presentation, patient and injury characteristics were prospectively collected by reviewing each patient’s electronic medical and surgical records, including gender, age, whether it was an isolated pelvic ring injury (no additional injuries sustained) and whether the patient sustained high-energy trauma.

Initial radiographs and CT scans were reassessed by two pelvic trauma surgeons (FIJ, KtD), and the injuries were classified according to the Young and Burgess classification system [1]. Patients with Young and Burgess classified LC1 fractures were identified for this study.

### Treatment

Treatment decisions were made according to standard practice, based on a combination of fracture pattern, pain, ability to mobilize, and patient preference. The radiographic scoring system was not included in the decision process. Whether the patient was treated non-operatively or operatively, and specific fixation methods were recorded.

### LC1 radiographic scoring system

**Fig. 1:**
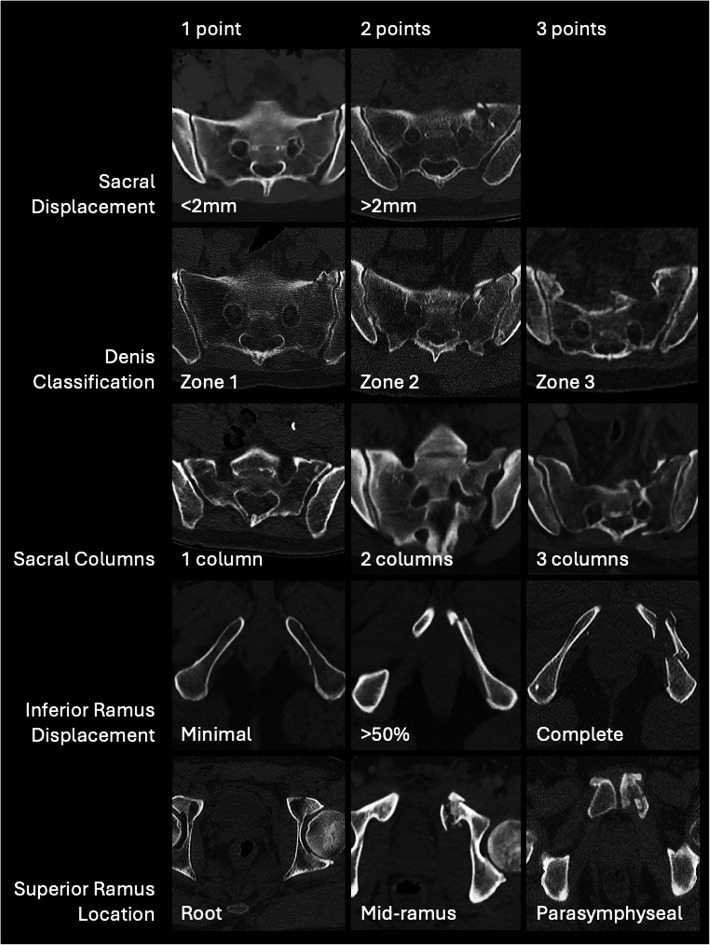
Lateral Compression 1 fracture scoring criteria examples

**Fig. 2:**
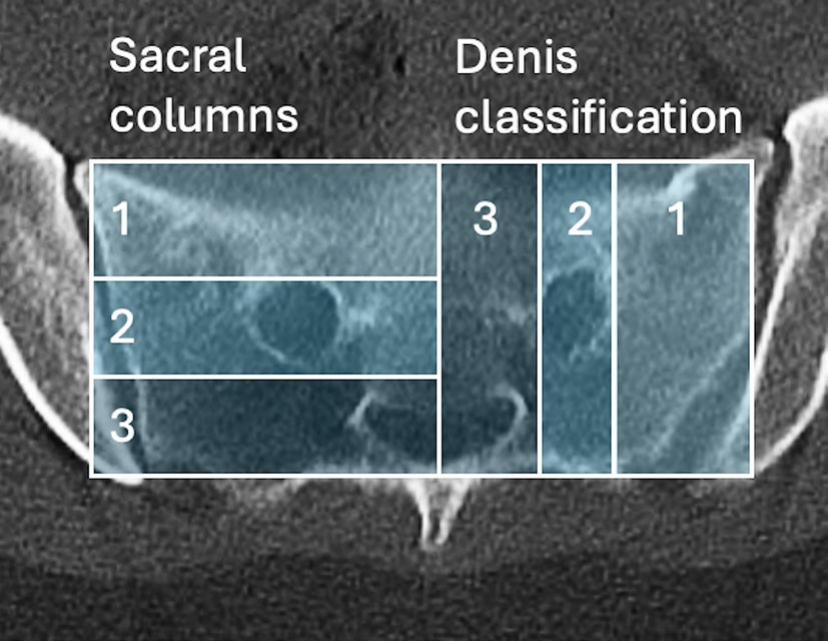
Sacral columns and Denis classification scoring explanation

The radiographic scoring system was applied to our patient population. As described by Beckman et al. [25], each CT was scored based on five radiographic parameters, resulting in a sum score that represents injury severity on a 5-to-14-point scale. The scoring criteria parameters are presented in Appendix 1, and examples are illustrated in Fig. 1. Figure 2 further explains the sacral columns and Denis classification scoring on a sacrum.

### Clinical outcomes

#### Delayed intervention due to non- or malunion

Treatment success was defined as no delayed intervention due to non- or malunion within one year after the injury. This outcome assessed whether non-operative treatment was appropriate. Reinterventions for operatively treated patients were also recorded.

#### Patient-reported functional status and health-related quality of life

Patient-reported outcome measures (PROMs) were prospectively collected at admission (pre-injury status), three months, six months, one year, and two years following the injury. PROMs were collected through digital questionnaires in RoQua, a secure online system linked to electronic patient files [31]. PROMs completed one or more years after the injury were used.

The Dutch Short Musculoskeletal Function Assessment (SMFA-NL) was used to evaluate patient-reported functional status. This questionnaire comprises 46 items, each answered on a five-point Likert scale. From these items, specific subscales can be derived, including lower extremity dysfunction (LED), difficulties with daily activities (ADL), and mental and emotional problems (MEP), all of which have demonstrated structural validity and reliability [32–34]. The individual item scores were summed and transformed into a total score ranging from zero to 100, with higher scores indicating better functional status.

To assess patient-reported health-related quality of life, the Dutch version of the EuroQol-5D 5L (EQ-5D) questionnaire was administered, evaluating five key dimensions: mobility, self-care, routine activities, pain/discomfort, and anxiety/depression [35]. Each dimension was answered on a five-point Likert scale, generating a utility score between zero and one, where higher scores indicate a better outcome. The EQ-5D is recognized as valid and reliable [36].

Age-matched normative data of the SMFA-NL (37) and the EQ-5D [35] were used to determine whether the patient recovered to the expected level. SMFA-NL normative data was further specified by gender. If the patient’s score reached the lower level of the 95% confidence interval of the normative data, the patient was considered recovered.

### Statistics

Statistical analyses were conducted using IBM SPSS software with a significance level of *p* < 0.05. Normally distributed data were presented as means and standard deviations (SD), while non-normally distributed data were presented as medians and interquartile ranges (IQR). Frequency was reported as n (%).

Patients were stratified into low (scores 5–6), intermediate (scores 7–9), and high (scores 10–14) score subgroups based on the Beckman et al. scoring system. Patient characteristics, treatment, PROMs and recovery rates were assessed within score subgroups.

Associations between PROMs, recovery rates, and treatment were analyzed using the Mann–Whitney U test for PROMs and the chi-square test for recovery.

A non-response analysis was performed to examine differences in patient and injury characteristics between responders and non-responders for PROMs.

Finally, associations between individual scoring components and PROMs were tested using the Mann–Whitney U test for sacral displacement (< 2 mm/ ≥ 2 mm) and Kruskal–Wallis tests for other components, with pairwise comparisons conducted for the latter.

## Results

### Study population

Patient and injury characteristics can be found in Table 1. Of the 203 patients presenting with an LC1 injury, 36 (18%) had scores 5–6, 105 (52%) had scores 7–9 and 62 (31%) had scores 10–14 (Table 2). No patients with scores of 5–6 were treated surgically, while 6% (n = 6) of patients with scores of 7–9 and 29% (n = 18) of patients with scores of 10–14 were treated operatively.

**Table 1:** Patient and injury characteristics

Radiographic LC1 scoring system	Scores 5–6	Scores 7–9		Scores 10–14	
	Conservative (n = 36)	Conservative (n = 99)	Operative (n = 6)	Conservative (n = 44)	Operative (n = 18)
Female, n (%)	21 (58%)	55 (56%)	3 (50%)	26 (59%)	6 (33%)
Age at the time of injury, mean (SD)/ median (IQR)	57 (SD 20)	55 (SD 22)	46 (IQR 36)	54 (SD 22)	56 (SD 15)
High-energy trauma, n (%)	23 (64%)	59 (60%)	5 (83%)	31 (71%)	11 (61%)
Fragility fracture*, n (%)	10 (28%)	31 (31%)	1 (17%)	12 (27%)	3 (16%)
Isolated pelvic ring injury, n (%)	12 (33%)	42 (43%)	3 (33%)	12 (27%)	5 (28%)
Associated acetabulum injuries, n (%)	5 (14%)	12 (12%)	2 (33%)	6 (14%)	3 (17%)
Associated lower extremity injuries, n (%)	2 (6%)	14 (14%)	1 (17%)	7 (16%)	1 (6%)
Deceased, < 30 days,n (%)	0	3 (3%)	0	4 (9%)	0
Deceased, < 1 year, n (%)	2 (6%)	5 (5%)	0	4 (%)	0
Anterior fixation**, n (%)	–	–	2 (33%)	–	6 (33%)
Posterior fixation***, n (%)	–	–	4 (77%)	–	4 (22%)
Anterior and posterior fixation****, n (%)	–	–	0	–	8 (44%)

^*^Fragility fractures were defined as injuries in patients aged 65 or older with a low-energy trauma mechanism

^**^ Anterior plate osteosynthesis

^***^Posterior only fixation: 2 with one sacroiliac screw, 2 with two sacroiliac screws, 3 with a trans-sacral screw, and 1 with both a trans-sacral screw and an additional sacroiliac screw

^****^ Both anterior and posterior fixation: 4 with anterior plate osteosynthesis and 1 sacroiliac screw, 1 with anterior plate osteosynthesis and 2 sacroiliac screws, 1 with anterior plate osteosynthesis and 2 trans-sacral screws, and 1 with anterior plate osteosynthesis, a trans-sacral screw, and a sacroiliac screw

**Table 2:** Components of the scoring system within the radiographic LC1 scoring system subgroup, stratified by treatment

	Low (scores 5–6)	Intermediate (scores 7–9)		High (scores 10–14)	
	Conservative (n = 36)	Conservative (n = 99)	Operative (n = 6)	Conservative (n = 44)	Operative (n = 18)
*Sacral displacement*					
< 2 mm, n (%)	34 (94%)	62 (63%)	0	10 (23%)	2 (11%)
≥ 2 mm, n (%)	2 (6%)	37 (37%)	6 (100%)	34 (77%)	16 (89%)
*Denis classification*					
Zone 1, n (%)	35 (97%)	57 (58%)	1 (17%)	10 (23%)	2 (11%)
Zone 2, n (%)	1 (3%)	39 (39%)	4 (67%)	32 (73%)	14 (78%)
Zone 3, n (%)	0	3 (3%)	1 (17%)	2 (5%)	3 (11%)
*Sacral columns*					
Column 1, n (%)	32 (89%)	41 (41%)	1 (17%)	2 (5%)	1 (6%)
Column 2, n (%)	4 (11%)	49 (50%)	2 (33%)	24 (55%)	3 (17%)
Column 3, n (%)	0	9 (9%)	3 (50%)	18 (41%)	14 (78%)
*Inferior ramus displacement*					
< 50%, n (%)	32 (89%)	55 (56%)	5 (83%)	10 (23%)	6 (33%)
50–99%, n (%)	4 (11%)	25 (25%)	1 (17%)	9 (21%)	2 (11%)
Complete, n (%)	0	19 (19%)	0	25 (57%)	10 (56%)
*Superior ramus location*					
Root, n (%)	31 (86%)	37 (37%)	4 (67%)	4 (9%)	1 (6%)
Mid-ramus, n (%)	5 (14%)	35 (35%)	1 (17%)	17 (39%)	0
Paraphyseal, n (%)	0	27 (27%)	1 (17%)	23 (52%)	17 (94%)

### The relation between the radiographic LC1 scoring system and treatment

**Fig. 3:**
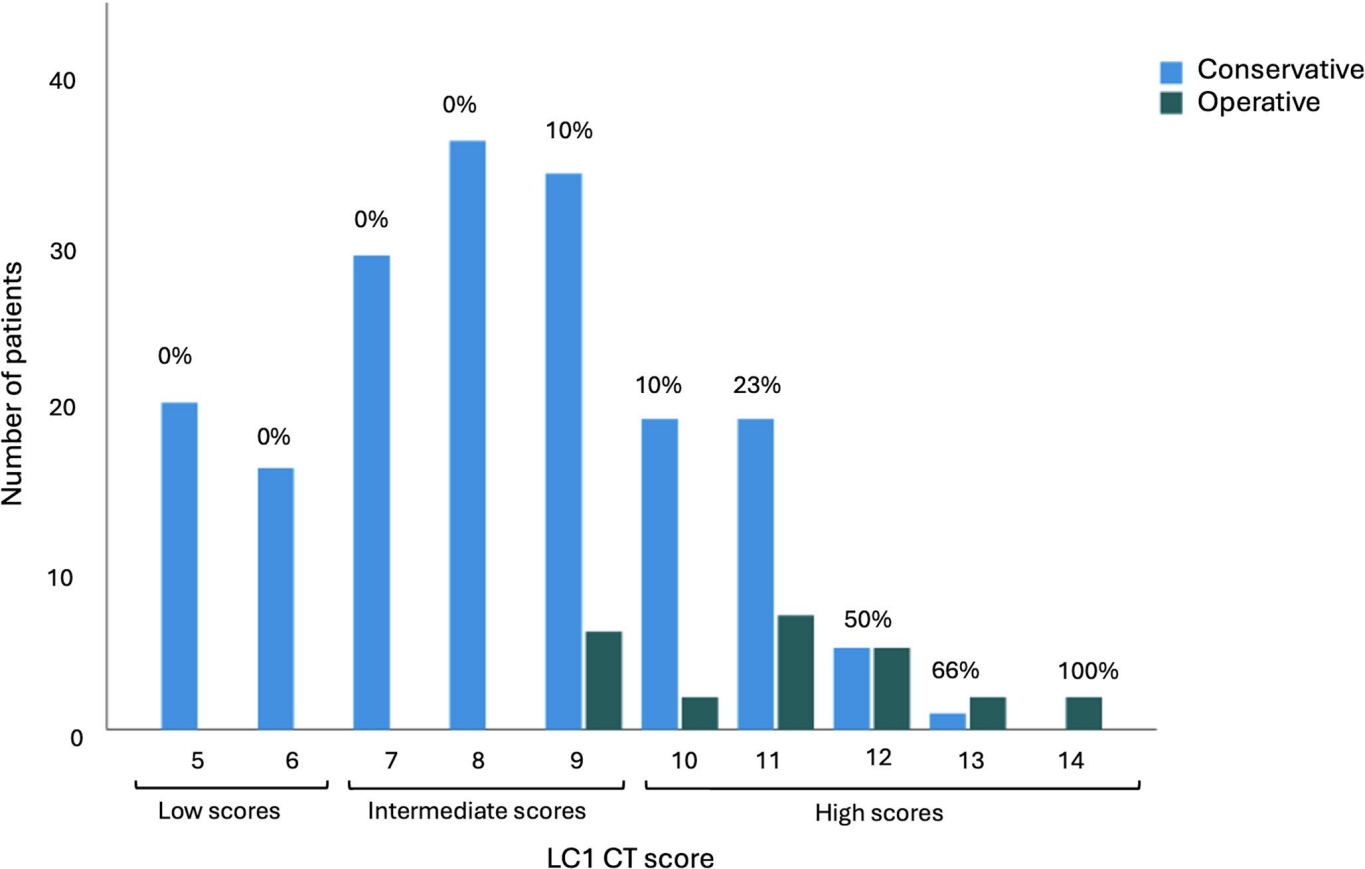
Split histogram for the radiographic LC1 scoring system for non-operatively and operatively treated patients. The low score subgroup consists of scores 5–6, the intermediate scores 7–9 and the high score subgroup 10–14. The percentages of patients treated operatively within each score are indicated in the figure

A histogram demonstrating the scores and the treatment decisions made is presented in Fig. 3. Of the patients with low scores (5–6), 100% (n = 36) were treated conservatively. In the intermediate subgroup (scores 7–9), 94% (n = 99) were treated conservatively. Lastly, in the subgroup with high scores (10–14), 71% (n = 44) were treated conservatively. Components of the scoring system for each subgroup can be observed in Table 2. The mean score for conservative patients was 8.2 (SD 1.9) and for operative patients 11.0 (SD 1.6).

### Delayed intervention

No non-operatively treated patients required delayed surgical intervention within at least one year due to non- or malunion. This was assessed in 165 patients who had reached the 1-year mark following the injury. Of the 24 operatively treated patients, four (17%) required later surgical reintervention to remove osteosynthesis material. In two patients, the anterior plate osteosynthesis was removed, one due to infection and one due to discomfort. A sacroiliac screw was removed in the other two patients, one due to pain and one due to neurological symptoms in the leg.

### Patient-reported functional status and health-related quality of life within score sub-groups

**Fig. 4:**
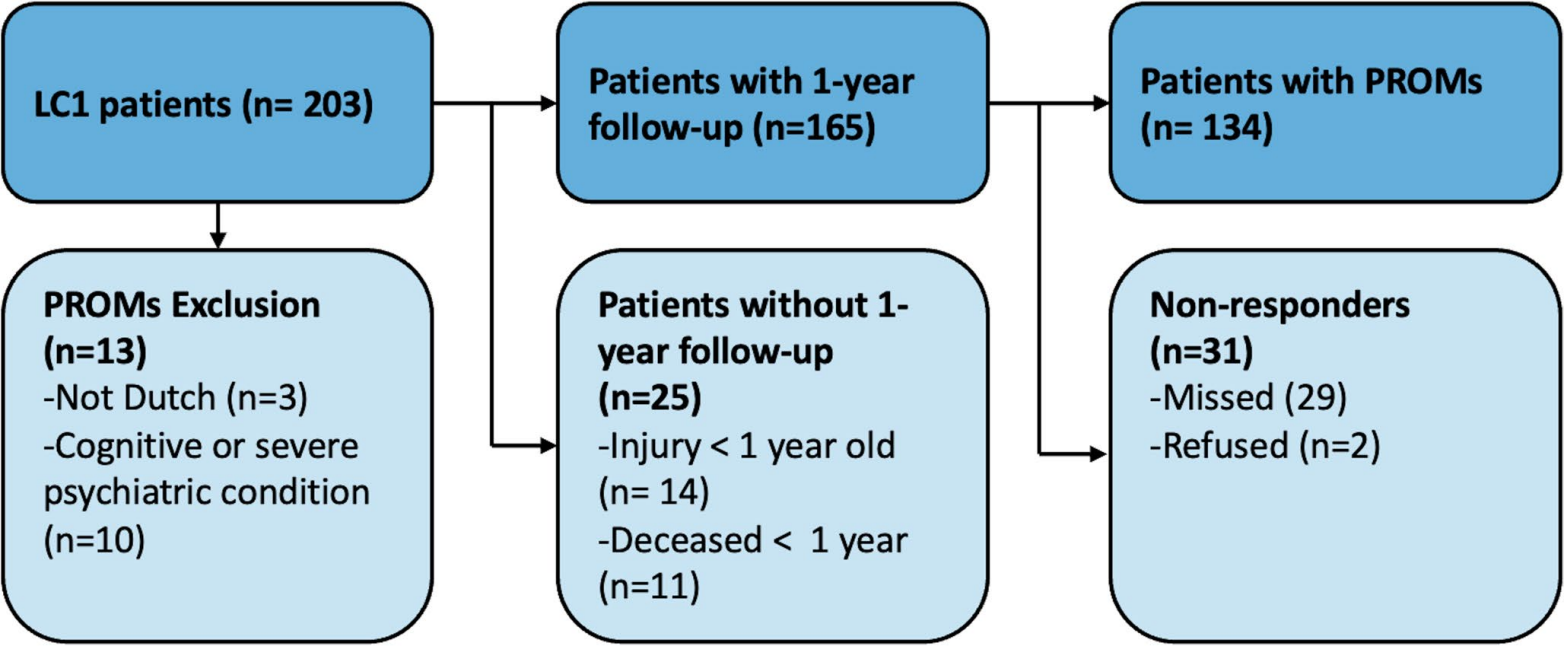
Flow chart for availability of patient-reported outcome measures (PROMs) for Lateral Compression 1 (LC1) patients

PROMs, presented in Table 3, were available for 82% (n = 134) of the eligible patients (Fig. 4). The median Follow-up time was 13 months (IQR: 12–24). A non-response analysis is available in Appendix 2, wherein the only significant difference was age.

**Table 3:** Patient-reported level of functional status and health-related quality of life within the radiographic LC1 scoring system subgroups, stratified by treatment

	Low (scores 5–6)	Intermediate (scores 7–9)			High (scores 10–14)		
	Conservative (n = 24)	Conservative (n = 59)	Operative (n = 5)	*p*-value*	Conservative (n = 30)	Operative (n = 16)	*p*-value*
*SMFA-NL*							
LED, median (IQR)	89.6 (77.1–100)	90.0 (71.9–100)	97.6 (75.5–99.0)	0.48	91.7 (78.6–96.4)	90.6 (75.5–99.5)	0.87
Recovered LED, n (%)	17 (71%)	37 (66%)	4 (80%)	0.42	23 (77%)	9 (56%)	0.14
ADL, median (IQR)	84.4 (63.4–93.1)	82.5 (63.7–95.6)	100 (49.5–100)	0.38	87.5 (67.8–97.5)	81.3 (62.8–99.5)	0.81
Recovered ADL, n (%)	13 (54%)	30 (51%)	3 (60%)	0.56	20 (67%)	8 (50%)	0.83
MEP, median (IQR)	82.8 (71.9–90.6)	81.3 (70.3–89.3)	96.9 (64.1–98.4)	0.39	82.8 (71.9–93.8)	89.1 (71.1–96.9)	0.38
Recovered MEP, n (%)	18 (75%)	39 (66%)	3 (60%)	0.51	22 (73%)	11 (69%)	0.50
EQ-5D, median (IQR)	0.84 (0.76–0.91)	0.81 (0.74–1.0)	1.0 (0.54–1.0)	0.34	0.85 (0.80–0.89)	0.87 (0.75–1.0)	0.39
Recovered EQ-5D, n (%)	12 (50%)	28 (47%)	3 (60%)	0.60	16 (53%)	12 (75%)	0.10

^*^The *p*-value represents the difference in the PROMs or recovery rate within the score subgroups for conservatively vs. operatively treated patients. Significance was set at *p* < 0.05

Dutch Short Musculoskeletal Function Assessment (SMFA-NL), lower extremity dysfunction subscale (LED), difficulties with daily activities subscale (ADL), mental and emotional challenges subscale (MEP), EuroQol-5D 5L (EQ-5D)

When comparing PROMs and the number of recovered patients between operatively and conservatively treated patients, tested separately in the intermediate and high score subgroups, no statistically significant differences were identified. Sub-group analyses of high- and low-energy traumas are presented in Appendix 3 and Appendix 4, which also showed no significant differences in outcomes between treatment groups.

### Relation between components of the radiographic LC1 scoring system and functional status and health-related quality of life

No associations were found between the various components of the scoring system and the PROMs (Appendix 5), nor while running pairwise comparisons.

## Discussion

LC1 pelvic ring injuries encompass a broad spectrum of fracture patterns and levels of instability [6–9], with no established consensus regarding the degree of instability or the optimal treatment [13, 15–18, 21–23]. To address this, Beckman et al. developed and validated a radiographic scoring system, ranging from 5 to 14, to specify fracture severity and help treatment decisions [25]. In this study, the validated Beckmann radiographic scoring system was applied to a prospective patient population in a level one trauma center. Patients with low scores (scores 5–6) and intermediate scores (scores 7–9) were predominantly treated conservatively, and with high scores (scores 10–14) either conservatively or operatively. No significant differences in outcomes were found between patients treated operatively or conservatively within the radiographic LC1 score subgroups, and the majority of patients made a full recovery. This implies that a non-operative treatment may be chosen for most patients, except for some with high scores.

The lack of consensus on the treatment of LC1 pelvic ring injuries stems from the wide variability in fracture patterns and instability within this classification. However, the radiographic scoring system aids in identifying the extremes. Injuries scoring five or six are relatively homogeneous, representing the most stable fracture types, with minor variation allowed within a one-point range.

Nonetheless, the scoring system still allows considerable variation within the intermediate subgroup (scores 7–9). Ultimately, the instability of an injury and the necessity of an operative approach depend on a combination of various components, and therefore, the dilemma of the spectrum of injury patterns remains. Regardless of the variation in the intermediate score subgroup, our study shows that this group can be successfully treated non-operatively, with no clinically important [38] or statistical differences in outcomes between operative and conservative patients. Thus, when feasible, surgeons should consider and attempt a non-operative approach for this group.

Furthermore, as 71% of patients in the high score subgroup were treated conservatively, with most making a full recovery, it should be considered that a non-operative treatment approach, including a period of limited weightbearing, may be a viable option for patients with high scores (scores 10–14). However, it should be noted that most patients (80%) with scores of 13 or 14 were operated on, as their injury patterns comprised a combination of the most unstable fracture patterns. Therefore, we propose splitting the high score subgroup into two groups: scores 10–12 and 13–14, as scores 10–12 may still have substantial variation in fracture pattern, while 13–14 is relatively homogenous (comparable to scores 5–6). Scores 13–14 should be treated operatively, and scores 10–12 should be considered for a conservative approach. Although specific reasons for an operative approach were not recorded during this study, the most frequently occurring fracture patterns identified in the operatively treated patients were sacral displacement (≥ 2 mm), an intraforaminal sacral fracture, a complete sacral fracture and a parasymphyseal superior ramus fracture. These patterns may reflect a higher degree of injury severity and could warrant a higher injury score, potentially helping to identify patients more likely to benefit from operative management in future classification systems. Inferior ramus displacement was less clearly related to operative treatment decisions.

Moreover, no associations between radiologic components of the LC1 scoring system, nor the score subgroups, and PROMs were identified. Livesey et al. found a significant difference in the frequency of late displacement in patients with scores more than nine compared to scores less than seven; however, the clinical implications were not investigated [27]. Additionally, it should be considered that all components of the scoring system are weighted equally in the radiographic LC1 scoring system, while the components may not be clinically proportional. It should also be noted that this scoring system primarily focuses on fracture location and not on morphological characteristics, such as comminution, obliquity, or transverse orientation, which are related to pelvic instability [39].

Several studies investigated outcomes in LC1 injuries treated operatively or conservatively [11, 16–20, 22–24]; however, treatment of these injuries remains controversial. A limitation of these studies is that they often consider LC1 injuries as one group and do not account for the variation of fracture patterns. Tucker et al. investigated patients with only stress-positive, minimally displaced LC1 injuries; however, only 43 patients were included [24]. Gaski et al. demonstrated acceptable functional outcomes in a subset of non-operatively treated LC1 injuries of “intermediate severity”; however, no comparison to operatively treated patients was performed [19]. As concluded by Khoury et al. [9], LC1 injuries as a single classification alone cannot describe these fractures or determine treatment. The added value of our study is that we provide outcomes for patients with moderate or combination fracture patterns by differentiating patients with clearly stable or unstable fracture patterns.

Our study has several strengths, including reporting treatment on a prospectively collected sample of 203 patients, representing a representative population treated at a level one trauma center. Of these patients, functional outcomes and health-related quality of life data were obtained for 134 patients, resulting in an 82% response rate. Moreover, the prospective collection of PROMs ensured standardized data acquisition, minimizing recall bias and enhancing reliability. Some limitations should be considered. Firstly, that PROMs were not available for all patients, potentially causing a response bias. That said, this is characteristic of a prospective study, and a non-response analysis showed no relevant differences. Another limitation, inherent to the use of PROMs, is that these measures are not specific to pelvic injury but rather reflect overall physical functioning and quality of life. To account for this, the proportion of patients with isolated pelvic injuries in each group was reported, which did not differ substantially between groups. Lastly, regarding the operatively treated patients, the limited sample size and variation in operative treatments restrict our ability to draw strong conclusions about their outcomes. No standard operative approach was implemented in our cohort, as injury patterns differ substantially between patients; treatment was therefore tailored individually and reported accordingly. This approach is consistent with the literature, which reports no consensus on the optimal management of these injuries [14]. Applying the radiographic LC1 scoring system in multiple centers with diverse treatment decisions and a higher number of operatively treated patients could further validate our results.

## Conclusion

The LC1 CT-based scoring system helps to clarify the spectrum of injury severity and assists in guiding treatment decisions. Patients with low (scores 5–6) and intermediate injury severity (scores 7–9), determined by multiple factors such as degree of sacral displacement, Denis classification, sacral column involvement, inferior ramus displacement, and superior ramus fracture location, can be treated conservatively with favorable clinical outcomes at one year follow-up. Patients with high injury severity (scores 10–14) can be treated either conservatively or operatively, with good outcomes in terms of delayed intervention and patient-reported functional status and health-related quality of life.

## Supplementary Information

Below is the link to the electronic supplementary material.

Supplementary Material 1

Supplementary Material 2

Supplementary Material 3

Supplementary Material 4

Supplementary Material 5

## Data Availability

No datasets were generated or analysed during the current study.
