# Supplementary material for: A validated CT-based scoring system for lateral compression type one pelvic ring injuries provides insight into the spectrum of injury severity and guides treatment decisions; a prospective study
Source: Eur J Orthop Surg Traumatol. 2026 Jan 22;36(1):82. doi: 10.1007/s00590-025-04619-4 (PMC12827294; doi:10.1007/s00590-025-04619-4)
Supplement: Supplementary file 1 — Supplementary Material 1 [file 590_2025_4619_MOESM1_ESM.docx]

***Appendix 1*** *Lateral Compression 1 fracture scoring criteria (25).*

| ***Parameter*** | ***Points*** |
| --- | --- |
| ***Sacral Displacement*** |  |
| *< 2 mm* | 1 |
| *≥ 2 mm* | 2 |
| ***Denis Classification*** |  |
| *Zone 1* | 1 |
| *Zone 2* | 2 |
| *Zone 3* | 3 |
| ***Sacral Columns*** |  |
| *1 column* | 1 |
| *2 columns* | 2 |
| *3 columns* | 3 |
| ***Inferior Ramus Displacement*** |  |
| *Minimal* | 1 |
| *>50%* | 2 |
| *Complete* | 3 |
| ***Superior Ramus Location*** |  |
| *Root* | 1 |
| *Mid-ramus* | 2 |
| *Parasymphyseal* | 3 |
