# Supplementary material for: A validated CT-based scoring system for lateral compression type one pelvic ring injuries provides insight into the spectrum of injury severity and guides treatment decisions; a prospective study
Source: Eur J Orthop Surg Traumatol. 2026 Jan 22;36(1):82. doi: 10.1007/s00590-025-04619-4 (PMC12827294; doi:10.1007/s00590-025-04619-4)
Supplement: Supplementary file 2 — Supplementary Material 2 [file 590_2025_4619_MOESM2_ESM.docx]

***Appendix 2*** *Non-response analysis*

|  | ***Responders (n=134)*** | ***Non-responders (n=31)*** | | ***P-value**** |
| --- | --- | --- | --- | --- |
| *Female, n (%)* | 75 (56%) | 18 (58%) |  | 0.50 |
| *Age at the time of injury, mean (SD)* | 55 (20) | 58 (26) |  | **0.002** |
| *High-energy trauma, n (%)* | 86 (63%) | 18 (62%) |  | 0.33 |
| *Fragility fracture*, n (%)* | 36 (27%) | 12 (39%) |  | 0.14 |
| *Isolated pelvic ring injury, n (%)* | 51 (38%) | 12 (39%) |  | 0.51 |
| *Associated acetabulum injuries, n (%)* | 20 (15%) | 2 (7%) |  | 0.17 |
| *Associated lower extremity injuries, n (%)* | 17 (13%) | 3 (10%) |  | 0.46 |
| *Beckmann score, mean (SD)* | 8.6 (2.2) | 8.2 (2.2) |  | 0.84 |

* Significance was set as a p-value of <0.05
