# Supplementary material for: A validated CT-based scoring system for lateral compression type one pelvic ring injuries provides insight into the spectrum of injury severity and guides treatment decisions; a prospective study
Source: Eur J Orthop Surg Traumatol. 2026 Jan 22;36(1):82. doi: 10.1007/s00590-025-04619-4 (PMC12827294; doi:10.1007/s00590-025-04619-4)
Supplement: Supplementary file 3 — Supplementary Material 3 [file 590_2025_4619_MOESM3_ESM.docx]

***Appendix 3*** *High-energy trauma mechanism subgroup analysis of patient-reported level of functional status and health-related quality of life within the radiographic LC1 scoring system subgroups, stratified by treatment.*

|  | ***Low (scores 5-6)*** | **Intermediate (*scores 7-9)*** | | | ***High (scores 10-14)*** | | |
| --- | --- | --- | --- | --- | --- | --- | --- |
|  | **Conservative (n=17)** | **Conservative (n=35)** | **Operative (n=4)** | **P-value*** | **Conservative (n=20)** | **Operative (n=10)** | **P-value*** |
| *SMFA-NL* |  |  |  |  |  |  |  |
| *LED, median (IQR)* | 95.8 (77.1-100) | 91.7 (75-100) | 97.9 (91.7-99.5) | 0.41 | 93.8 (78.6-96.4) | 92.7 (76.6-98.4) | 0.58 |
| *Recovered LED, n (%)* | 11 (65%) | 25 (71%) | 4 (100%) | 0.56 | 16 (80%) | 6 (60%) | 0.38 |
| *ADL, median (IQR)* | 87.5 (58.1-96.3) | 90.0 (65.0-96.3) | 100 (73.8-100) | 0.09 | 90 (80-98.4) | 88.1 (61.6-97.8) | 0.52 |
| *Recovered ADL, n (%)* | 10 (59%) | 18 (51%) | 3 (75%) | 0.61 | 15 (75%) | 6 (60%) | 0.43 |
| *MEP, median (IQR)* | 81.25 (62.5-92.2) | 78.1 (62.5-90.9) | 96.9 (68.8-99.2) | 0.22 | 84.4 (75-93.7) | 87.5 (64.1-96.9) | 0.93 |
| *Recovered MEP, n (%)* | 12 (71%) | 21 (60%) | 3 (75%) | 1.00 | 15 (75%) | 6 (60%) | 0.43 |
| *EQ-5D, median (IQR)* | 0.82 (0.74-0.96) | 0.88 (0.74-1.0) | 1.0 (0.83-1.0) | 0.21 | 0.85 (0.81-0.89) | 0.87 (0.82-1.0) | 0.38 |
| *Recovered EQ-5D, n (%)* | 7 (41%) | 19 (54%) | 3 (75%) | 0.62 | 11 (55%) | 8 (80%) | 0.25 |

*The p-value represents the difference in the PROMs or recovery rate within the score subgroups for conservatively vs. operatively treated patients. Significance was set at p<0.05

Dutch Short Musculoskeletal Function Assessment (SMFA-NL), lower extremity dysfunction subscale (LED), difficulties with daily activities subscale (ADL), mental and emotional challenges subscale (MEP), EuroQol-5D 5L (EQ-5D)
