# Supplementary material for: A validated CT-based scoring system for lateral compression type one pelvic ring injuries provides insight into the spectrum of injury severity and guides treatment decisions; a prospective study
Source: Eur J Orthop Surg Traumatol. 2026 Jan 22;36(1):82. doi: 10.1007/s00590-025-04619-4 (PMC12827294; doi:10.1007/s00590-025-04619-4)
Supplement: Supplementary file 4 — Supplementary Material 4 [file 590_2025_4619_MOESM4_ESM.docx]

***Appendix 4*** *Low-energy trauma mechanism subgroup analysis of patient-reported level of functional status and health-related quality of life within the radiographic LC1 scoring system subgroups, stratified by treatment.*

|  | ***Low (scores 5-6)*** | **Intermediate (*scores 7-9)*** | | | ***High (scores 10-14)*** | | |
| --- | --- | --- | --- | --- | --- | --- | --- |
|  | **Conservative (n=7)** | **Conservative (n=24)** | **Operative (n=1)** | **P-value*** | **Conservative (n=10)** | **Operative (n=6)** | **P-value*** |
| *SMFA-NL* |  |  |  |  |  |  |  |
| *LED, median (IQR)* | 89.6 (77.1-100) | 83.3 (67.1-94.8) | 68.8 (68.8-68.8) | 0.41 | 83.3 (68.3-91.1) | 83.3 (58.3-100) | 0.66 |
| *Recovered LED, n (%)* | 6 (86%) | 12 (50%) | 0 | 0.48 | 7 (70%) | 3 (50%) | 0.61 |
| *ADL, median (IQR)* | 78.8 (70.0-91.3) | 76.9 (59.4-93.1) | 33.8 (33.8-33.8) | 0.10 | 74.4- (53.4-91.9) | 70.0 (57.2-100) | 0.74 |
| *Recovered ADL, n (%)* | 3 (43%) | 12 (50%) | 0 | 0.48 | 5 (50%) | 2 (33%) | 0.63 |
| *MEP, median (IQR)* | 84.4 (71.9-87.0) | 82.8 (75.0-89.4) | 68.8 (68.8-68.8) | 0.20 | 76.6 (63.2-90.6) | 89.1 (70.3-100) | 0.25 |
| *Recovered MEP, n (%)* | 6 (86%) | 18 (75%) | 0 | 0.22 | 7 (70%) | 5 (83%) | 1.00 |
| *EQ-5D, median (IQR)* | 0.85 (0.76-0.91) | 0.81 (0.69-0.89) | 0.31 (0.31-0.31) | 0.13 | 0.83 (0.70-1.0) | 0.87 (0.54-1.0) | 0.78 |
| *Recovered EQ-5D, n (%)* | 5 (71%) | 9 (38%) | 0 | 1.00 | 5 (50%) | 4 (67%) | 0.63 |

Dutch Short Musculoskeletal Function Assessment (SMFA-NL), lower extremity dysfunction subscale

*The p-value represents the difference in the PROMs or recovery rate within the score subgroups for conservatively vs. operatively treated patients. Significance was set at p<0.05

Dutch Short Musculoskeletal Function Assessment (SMFA-NL), lower extremity dysfunction subscale (LED), difficulties with daily activities subscale (ADL), mental and emotional challenges subscale (MEP), EuroQol-5D 5L (EQ-5D)
