# Supplementary material for: A validated CT-based scoring system for lateral compression type one pelvic ring injuries provides insight into the spectrum of injury severity and guides treatment decisions; a prospective study
Source: Eur J Orthop Surg Traumatol. 2026 Jan 22;36(1):82. doi: 10.1007/s00590-025-04619-4 (PMC12827294; doi:10.1007/s00590-025-04619-4)
Supplement: Supplementary file 5 — Supplementary Material 5 [file 590_2025_4619_MOESM5_ESM.docx]

***Appendix 5*** *Relation between components of the radiographic LC1 scoring system and patient-reported outcome measurement*

| *P-value** | ***Sacral Displacement*** | ***Denis classification*** | ***Sacral columns*** | ***Interior ramus displacement*** | ***Superior ramus fracture location*** |
| --- | --- | --- | --- | --- | --- |
| ***SMFA-NL ***** |  |  |  |  |  |
| *LED* | 0.65 | 0.43 | 0.24 | 0.93 | 0.23 |
| *ADL* | 0.87 | 0.40 | 0.54 | 0.94 | 0.65 |
| *MEP* | 0.87 | 0.42 | 0.51 | 0.47 | 0.47 |
| ***EQ-5D*** | 0.84 | 0.36 | 0.47 | 0.64 | 0.71 |

* Significance was set as a p-value of <0.05

**Dutch Short Musculoskeletal Function Assessment (SMFA-NL), lower extremity dysfunction (LED), difficulties with daily activities (ADL), mental and emotional challenges (MEP), EuroQol-5D 5L (EQ-5D)
